# Devon and Exeter Hospital

**Published:** 1892-10-15

**Authors:** 


### WITHIN THE HOSPITALS.

### DEVON AND EXETER HOSPITAL.

By Our Special Commissioner.

This institution is typical of the best class of county infirmary of forty years ago. Situated in a cathedral city it has pursued the even tenor of its way without material change, and the improvements of modern times do not appear to have permeated the minds of the governing body. It possesses several excellent points, but the furniture, interior decorations, and fittings show where a large sum might be usefully spent in the best interests of all concerned. The inspections we have been making of late have convinced us that the hospital economists, often met with on our country hospital boards, might usefully exert their influeuce to prevent the expenditure of money upon new buildings. Those gentlemen should, however, at the same time advocate the annual outlay of a considerable sum upon the interior arrangements and fittings, so as to secure that every part of the existing building is brought up to date by being made replete with every modern appliance and with perfectly modern furniture. Go where he may, the eye of the visitor finds evidence of the urgent need which exists for an immediate re-decoration, re-fitting, and re-furnishing of the Devon and Exeter Hospital. Some of the beds are of a pattern so old as to belong to a past generation, and the need for decorative repair is everywhere apparent. As an illustration of deterioration, we may point out that in one of the wards in the Hertford wing the porcelain bath has been painted, so that a bath which originally presented and should still present an inviting appearance, has become exactly the reverse. We would suggest that it would well repay the manufacturers to offer to replace the old bath with a new and cleanly porcelain bath, in order that they may retain the former in their show-rooms as an example of how not to do it. A considerable sum ought at once to be voted to enable the authorities to replace the existing baths, slop sinks, &c. Exeter has an ably-conducted local press, and we would ask the editors to send some qualified person to go over the Devon and Exeter Hospital, to see whether it is not precisely true that owing to much wear and tear the appearance of the wards and offices is far behind that of a modern poor law infirmary. The new nurses' home gives evidence that the Committee understand how to furnish wisely and well. We hope, therefore, now that attention has been drawn to the internal decorations of this important county hospital, that steps will speedily be taken in the direction of entire renovation. New buildings are not required at Exeter, but re-fitting and re-furnishing is urgently called for, unless the citizens of Exeter are content to allow their hospital to take a second place when compared with similar institutions throughout the country. We would suggest that they should visit Salisbury, and see what the Committee of that institution have been able to do in the way of making an old building replete with every modern improvement by which the efficiency can be increased.

### Special Features.

The Devon and Exeter Hospital was, we believe, the first institution to recognise the desirability of separation wards for the treatment of severe cases after operation. In connection with the operation theatre a number of single-bedded rooms have been erected, wherein cases of this description can be treated under the most favourable conditions. This feature at Exeter has attracted from time to time considerable notice, and it has been adopted at some of the best British and foreign hospitals. We noticed in the operation theatre a number of old sponges placed ready for use. We hoped that the dangers of using old sponges had been sufficiently driven home to prevent the practice in any British hospital at the present time. It appears to be the practice at Exeter to devote some of the beds to chronic cases. There are upwards of twenty chronic cases in the hospital, some of which have resided there from March 8th, 1888. In the case of county infirmaries, where there is no great pressure upon beds, it would be of immense importance to the poor that a certain number of them should be devoted, as at Exeter, to the accommodation of chronic cases. They include various hip and other joint cases, cases of dyspepsia, lupus, and chorea, and cerebral and gastric cases. Contrary to the experience at most hospitals, the out-patients at Exeter appear to be falling off. Thus, the number of out-patients decreased from 2,508 in 1891 to 2,333 in 1892, and of those out-patients treated 1,261 had previously been in-patients. We observe, further, that the amounts collected on Hospital Sunday have steadily decreased. The sum received in 1892 (viz., £791) is very low, being nearly £100 less than the average for the last ten years, and nearly £600 less than the amount raised in the first year of the fund. Exactly the same thing happened in London, until the Council of the Hospital Sunday Fund called public attention to the matter and enlisted the warm sympathy of the clergy and ministers of all denominations. If each preacher would make it his business next Hospital Sunday to visit the Devon and Exeter Hospital so as to see for himself the good work it is doing, and to ascertain its needs and requirements, we make no doubt that the amount collected on Hospital Sunday might easily be increased to £1,500 per annum. At Exeter there is a library and a museum, the former of which contains many volumes of interest and value, and we hope steps may be taken to keep this library up to date, and to maintain it in efficiency. Libraries at the County Hospitals fulfilled a most useful function in the past, many of them, like that at Exeter, contain rare volumes of great interest, and it would be a pity if the want of a little vigorous attention should allow them to diminish or disappear.

### Drunk or Dying.

The newspapers from time to time contain paragraphs under this head which relate to cases of mistaken diagnosis which have been refused admission by hospital authorities, and so found their way to the police cells. At Exeter a special ward is provided on the ground floor, which is set apart for the reception of delirium tremens, and of doubtful cases. We have often pointed out that every metropolitan and all large and well-administered provincial hospitals should contain a special ward of this description. The resident medical staff are necessarily young men, and from time to time it may happen that a Resident may have to deal with a doubtful case within a week of his appointment to office. It is not reasonable to expect that the younger members of the profession can spontaneously acquire wide experience, and so it happens in the absence of an "emergency ward" that mistakes necessarily occur. When they do occur great blame is usually meted out to the young medical practitioner, whereas the hospital authorities ought in reality to be made entirely responsible unless they have provided a special ward, and issued instructions that all doubtful cases shall be admitted to it, and retained there for twenty-four hours at least, so as to enable a correct diagnosis to be made. The fact that the medical staff of the Exeter Hospital have provided such a ward is greatly to their credit, and deserves full recognition.

### Nurses' Home.

The new nurses' home has been tastefully furnished, and the recreation room is a most comfortable apartment. We observed, however, that there is no library at present for the nurses, and we hope that some of the residents of Exeter will take an interest in the matter and see that a well-assorted collection of books is speedily provided for the use of the nurses. It is a great pity that a painted iron bath, in lieu of a porcelain one, should have been fixed in the nurses' home. We were sorry, too, to observe, that the architect has provided no fireplaces, as he should have done, in many of the nurses' rooms, and that those of them which contain two beds are not provided with screens. Experience shows that it is, on the highest grounds, most desirable to encourage each nurse to respect herself by giving her, wherever possible, a separate bed-room, and when this is not possible, to at any rate provide that her share of any space allotted to her for sleeping purposes shall be self-contained and private. These, though relatively small, are important matters, which we commend to the attention of the authorities. It appears that the plan of sending out nurses to private cases from the hospital has commended itself to the people of Exeter, whilst it has proved highly profitable to the hospital authorities. Thus the receipts from private nursing, which amounted to £59 in 1891, increased to £373 in 1892. This gives the committee the command of a considerable income, which ought to be devoted in the first instance to the welfare of the workers, who have earned the money. For many years apparently it has been the practice at Exeter to grant annuities for life to certain members of the staff. Thus, the accounts show a payment of £69 to four nurses, and one porter "since deceased." It is likely that the number of nurses engaged in private work will be increased, and the committee will soon have to face the provision of sick pay during the time the nurses are ill. For this and for other reasons, we would commend to their attention the scheme of the Royal National Pension Fund, as if they decide to affiliate, they will be able to secure sick pay and pensions for their nurses on a much cheaper and more efficient plan than they can otherwise hope to do.

